# Versican but not decorin accumulation is related to malignancy in mammographically detected high density and malignant-appearing microcalcifications in non-palpable breast carcinomas

**DOI:** 10.1186/1471-2407-11-314

**Published:** 2011-07-26

**Authors:** Spyros S Skandalis, Vassiliki T Labropoulou, Panagiota Ravazoula, Eleni Likaki-Karatza, Katalin Dobra, Haralabos P Kalofonos, Nikos K Karamanos, Achilleas D Theocharis

**Affiliations:** 1 Laboratory of Biochemistry, Department of Chemistry, University of Patras, Rio 26504, Greece; 2 Ludwig Institute for Cancer Research, Uppsala Branch, Box 595, 75124 Uppsala, Sweden; 3 Clinical Oncology Laboratory, Division of Oncology, Department of Medicine, University Hospital of Patras, Patras Medical School, Rio 26504, Greece; 4 Department of Pathology, University Hospital of Patras, Rio 26504, Greece; 5 Department of Radiology, University Hospital of Patras, Rio 26504, Greece; 6 Department of Laboratory Medicine, Division of Pathology, Karolinska Institute, F-46 Huddinge University Hospital, SE-14186 Stockholm, Sweden

**Keywords:** proteoglycans, versican, decorin, mammographic density, malignant-appearing microcalcifications, non-palpable breast carcinomas

## Abstract

**Background:**

Mammographic density (MD) and malignant-appearing microcalcifications (MAMCs) represent the earliest mammographic findings of non-palpable breast carcinomas. Matrix proteoglycans versican and decorin are frequently over-expressed in various malignancies and are differently involved in the progression of cancer. In the present study, we have evaluated the expression of versican and decorin in non-palpable breast carcinomas and their association with high risk mammographic findings and tumor characteristics.

**Methods:**

Three hundred and ten patients with non-palpable suspicious breast lesions, detected during screening mammography, were studied. Histological examination was carried out and the expression of decorin, versican, estrogen receptor α (ERα), progesterone receptor (PR) and c-erbB2 (HER-2/neu) was assessed by immunohistochemistry.

**Results:**

Histological examination showed 83 out of 310 (26.8%) carcinomas of various subtypes. Immunohistochemistry was carried out in 62/83 carcinomas. Decorin was accumulated in breast tissues with MD and MAMCs independently of the presence of malignancy. In contrast, versican was significantly increased only in carcinomas with MAMCs (median ± SE: 42.0 ± 9.1) and MD (22.5 ± 10.1) as compared to normal breast tissue with MAMCs (14.0 ± 5.8), MD (11.0 ± 4.4) and normal breast tissue without mammographic findings (10.0 ± 2.0). Elevated levels of versican were correlated with higher tumor grade and invasiveness in carcinomas with MD and MAMCs, whereas increased amounts of decorin were associated with *in situ *carcinomas in MAMCs. Stromal deposition of both proteoglycans was related to higher expression of ERα and PR in tumor cells only in MAMCs.

**Conclusions:**

The specific accumulation of versican in breast tissue with high MD and MAMCs only in the presence of malignant transformation and its association with the aggressiveness of the tumor suggests its possible use as molecular marker in non-palpable breast carcinomas.

## Background

Breast carcinoma is considered to be one of the main causes of cancer mortality. Assessment of the risk of development of invasive breast cancer has become a significant problem. In the last decade, screening programs have been intensified since mammographic screening significantly contributes on breast cancer mortality [1,2]. The major aim of these programs is the detection of breast carcinomas in earlier and probably better curable stage [3]. In the past 20 years, concomitant with the wide use of screening mammography, the incidence of ductal carcinoma *in situ *(DCIS) has risen dramatically, in asymptomatic women to 20-25% of all screening detected breast cancers [4]. Therefore, the mammographically diagnosed non-palpable breast carcinomas are increasingly considered as a unique entity of major clinical interest. Non-palpable breast carcinomas consists a heterogeneous group of lesions with variable findings and different prognosis. Mammographically detected density is a risk factor for breast cancer and is attributed to alterations in the composition of breast tissue [5,6]. Previous studies seeking to understand the biological basis of mammographic density (MD) have focused on associations with epithelial and stromal changes [7,8]. Another mammographic finding of higher risk than tissue density for breast cancer is malignant-appearing microcalcifications (MAMCs), which are associated with *in situ *and invasive breast carcinomas in asymptomatic women [9]. MAMCs are the primary indication for approximately 50% of the breast biopsies carried out for non-palpable mammographic abnormalities, although they do not always represent malignancy [10].

A wide range of prognostic markers have been proposed for non-palpable breast carcinomas. The clinically available markers such as histological type, size, auxiliary node involvement and cytological grading are not sufficient, considering the biological complexity of this clinical entity [11]. Several biological markers such as estrogen receptor alpha (ERα), progesterone receptor (PR), and the ErbB family of receptor tyrosine kinases have been evaluated by means of immunohistochemistry in non-palpable breast carcinomas and found to correlate with mammographic findings of higher risk such as MAMCs [12,13]. Estrogens contribute to the initiation and promotion of cancer through triggering the proliferation of breast epithelium and stroma. Consequently they increase the changes of mutation in rapidly proliferating epithelium and those effects accumulate with increasing cumulative exposure to estrogens [14]. The over-expression of c-erbB2 (HER-2/neu) is associated with more aggressive tumor behavior [15].

Although breast cancer is a direct manifestation of alterations in the expression of multiple genes and cellular pathways within the cancer cell, it is now recognized that perturbations in stromal-epithelial interactions also influence tumorigenesis and progression through direct effects on growth factor-induced signaling pathways and indirect effects mediated through cell adhesion and structure [8,16,17].

Several studies have demonstrated abnormal expression of the matrix-secreted proteoglycans versican and decorin in various cancer types such as prostate [18,19], breast [20,21], gastric [22], colorectal [23,24], ovarian [25], pancreatic [26], laryngeal [27,28] and testicular tumors [29]. Versican is synthesized mainly by stromal cells and is capable to regulate tumor cell growth and motility. Versican may facilitate the local expansion of cancer cells and, subsequently, the invasion and formation of distant metastases by decreasing cell-matrix adhesion, sufficient to promote cancer cell migration through the extracellular matrix [30-32]. This notion is supported by observations that relapse in women with stage I node-negative breast cancer is related to the level of versican accumulated in peritumoral stroma [21] and the increased levels of peritumoral versican are also predictive of poor prognosis in patients with early-stage prostatic cancer [18]. In contrast, decorin, which is mainly over-expressed by activated fibroblasts in various cancer types, is considered to be a tumor suppressor proteoglycan [18,22-24,26-29,32]. Others have previously shown that matrix proteoglycans lumican and decorin are abundant components of breast tissue stroma and that altered expression of lumican and decorin is associated with tumor progression and outcome [33-35]. These proteoglycans are important for stromal integrity through their implication in fibrillar collagen cross linking. Decorin is also a powerful regulator of growth factor-mediated signaling [32].

We therefore wished to examine the relationship between mammographic findings suggestive of malignancy in non-palpable breast carcinomas and the expression of stromal proteoglycans versican and decorin, to establish whether the increased risk attributed to these findings might reflect stromal alterations. The expression of both proteoglycans was correlated with tumor characteristics and established biomarkers of the disease to evaluate their implication in breast cancer biology. Both proteoglycans were accumulated in MD and MAMCs although decorin was most likely associated with tissue fibrosis and matrix deposition. Versican was specifically accumulated in MD and MAMCs in the presence of malignant transformation and was significantly correlated with increased tumor grade and invasiveness. The over-expression of both proteoglycans is associated with the presence of ERα and PR in tumor cells in MAMCs.

## Materials and methods

### Patient population

This is a retrospective, single-center study conducted at the University Hospital of Patras, Greece. The conduct of the study was approved by the institutional review board of Patras Medical School and written informed consent was obtained from the patients. Between 1989 and 2002, 310 patients with non-palpable suspicious breast lesions detected during screening mammography were evaluated. The median age of the patients was 55 years, (range, 35-74 years) and none of the patients had previously been screened or shown clinical signs of breast disease.

### Mammography

The standard craniocaudal and lateral views were carried out in all patients. Mammographic findings requiring further exploration with breast biopsy were considered the following: (1) microcalcifications; (2) mass with or without microcalcifications; (3) architectural distortion with or without microcalcifications; and (4) asymmetric density with the greater diameter < 1 cm with or without microcalcifications [10]. Based on published observations, we evaluated MAMCs according to their shape (e.g. pleomorphic, irregular, fragmented, casting), density (highly variable), distribution (e.g. clustered) and size (highly variable) [36].

### Breast lesion localization

All patients with suspicious and highly suggestive of malignancy non-palpable breast lesions classified as BI-RADS 4 and BI-RADS 5 underwent pre-operative mammographically guided breast lesion localization with a Kopan breast localization needle (19G, 9 cm length) [37].

### Immunohistochemistry

Histological examination of the specimens showed 83/310 carcinomas. Immunohistochemical analysis was carried out in 62 out of 83 carcinomas, on the basis of tissue availability, 9 normal breast tissues, 14 breast tissues with high MD and 14 breast tissues with MAMCs without pathologic findings. MAMCs was the prominent finding in 48 cases and 14 cases were characterized by increased MD without microcalcifications. Tissue sections were obtained from the files of the Department of Pathology in the University Hospital of Patras. A panel of antibodies was employed for versican (2-B-1 Seikagaku), decorin (6-B-6 Seikagaku), ERα (6F11 Novocasta), PR (1A6 Novocasta), c-erbB2 (CB11 Biogenex). Avidin-biotin-peroxidase complex method (Dako Co., Copenhagen, Denmark) and microwave antigen retrieval was carried out. Tissue samples were fixed in 10% buffered formalin and embedded in paraffin. Serial 5- μm sections were taken and deparaffinized with xylene and dehydrated with 98% ethanol. The sections were treated with 1 U/ml chondroitinase ABC for 15 min at 37°C to detach glycosaminoglycan side chains from the protein core for versican and decorin staining. Endogenous peroxidase activity was quenched with 3% hydrogen peroxide for 5 min at room temperature. Non-specific protein binding of the antibodies was blocked by incubation with 3% normal swine serum in PBS for 20 min at room temperature. Slides were incubated with the antibodies diluted in PBS containing 1% normal swine serum for 1 h at room temperature. The resulting antigen- antibody complexes were visualized by 30 min incubation at room temperature, using appropriate biotinylated secondary antibodies diluted 1:200 and the avidin-biotin-peroxidase technique (Dako Co., Copenhagen, Denmark). The staining was developed with 3, 3-diaminobenzidine/hydrogen peroxide for 5 min at room temperature and slides were counterstained with haematoxylin. A positive tissue control and a negative reagent control (without primary antibody) were run in parallel. The level of decorin and versican expression was assessed by semiquantitative scoring devised by Alowami et al., [38] which includes (i) the overall percentage of the tissue section stained positive (0-100%), and (ii) the signal intensity (4-point scale) for each proteoglycan. The scoring used for the 4-point scale was: 1, negative or very weak staining; 2, weak positive; 3, moderate positive; 4, strong positive. The semiquantitative immunohistochemical (IHC) score for the expression level for each proteoglycan was provided by the multiplication of the percentage (0-100) of the tissue section staining positive by the factor (1-4) corresponding to the staining intensity of the tissue section. Positivity scoring for ERα, PR and c-erbB2 was carried out as described previously [12]. Three independent researchers randomly evaluated the specimens using this method.

### Statistical analysis

Data were analysed using GraphPad Prism (Version 3.0; GraphPad Software Inc., San Diego, CA, USA). Statistical analyses were performed using the Mann-Whitney *U*-test, Kruskal-Wallis Anova by ranks and linear regression analysis (Pearson). All tests were two-tailed and statistical significance was set at *P *< 0.05.

## Results

### Mammographic findings

**Figure 1:**
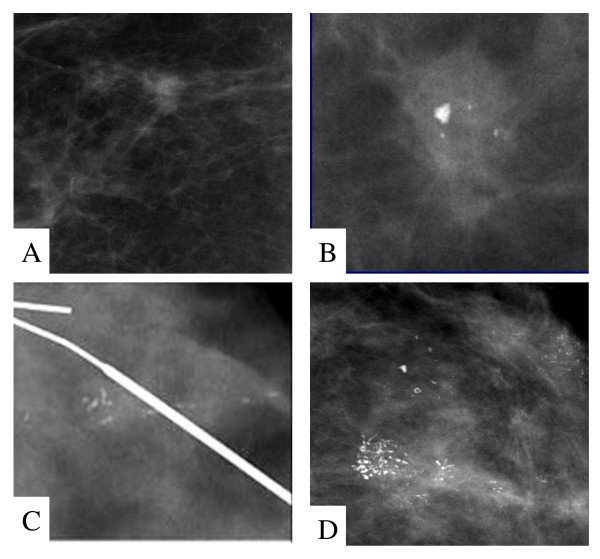
**Representative mammographic images of non-palpable breast carcinomas**. (A), High density with indistinct margins classified as BI-RADS 4. (B), High density with indistinct margins and microcalcifications classified as BI-RADS 4. (C), Linear microcalcifications, BI-RADS 4 and (D), pleomorphic multifocal calcifications, BI-RADS 4.

Three hundred and ten patients with suspicious and highly suggestive of malignancy mammographically found non-palpable breast lesions, classified as BI-RADS 4 and BI-RADS 5, who underwent mammographically guided needle excision biopsy were evaluated. Eighty-three out of 310 (26.8%) non-palpable breast lesions proved histologically to be carcinomas. Histological subtypes were 46/83 ductal invasive carcinomas (55.5%), 31/83 ductal *in situ *carcinomas (37.3%) and 6/83 lobular invasive carcinomas (7.2%). MAMCs as an isolated finding or in combination with a mass, density or distortion were detected in 65 out of 83 (78.3%) patients with non-palpable breast carcinoma (Table 1). Immunohistochemistry was performed in 62/83 carcinomas on the basis of tissue availability, 9 normal breast tissues, 14 breast tissues with high MD and 14 breast tissues with MAMCs without pathologic findings. The median age of the patients was 58 years (range, 41-74 years). Non-palpable breast carcinomas were divided in two groups according to their mammographic findings, one which is characterized by the presence of increased MD, mass and/or distortion (14/62) (Figure 1A) and the other characterized by the presence of MAMCs as an isolated finding or in combination with a mass, density or distortion (48/62) (Figure 1B-D). The expression of proteoglycans was evaluated in breast carcinomas and compared to normal breast tissues with identical mammographic findings.

**Table 1:** Mammographic appearance of histologically proven non-palpable breast carcinomas

Histology/Mammography	Invasive ductal carcinoma	*In situ *ductal carcinoma	Invasive lobular carcinoma	Total
Microcalcifications	20	19	1	40
Density, mass or distortion	12	3	3	18
Density, mass or distortion and microcalcifications	14	9	2	25
Total	46	31	6	83

### Decorin and versican expression and association with mammographic appearance

**Figure 2:**
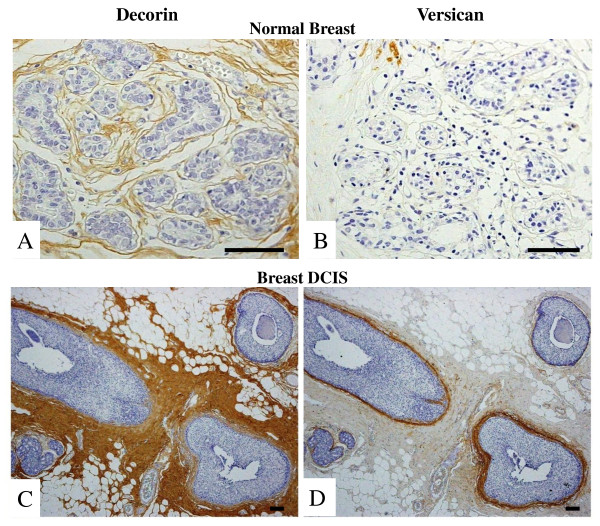
**Immunohistochemical localization of decorin and versican in normal breast and breast carcinomas**. Normal breast tissue (A and B) and non-palpable breast DCIS (C and D) stained for decorin (A and C) and versican (B and D). Bars, 100 μm.

**Figure 3:**
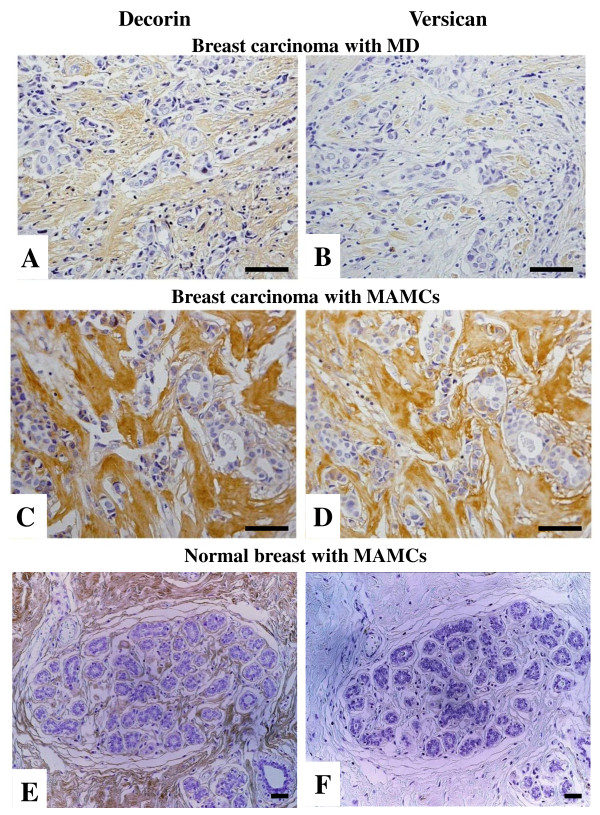
**Stromal deposition of decorin and versican in normal breast and breast carcinomas with MD and MAMCs**. Non-palpable breast carcinomas associated with high MD (A and B), mammographically found MAMCs (C and D) and normal breast tissue associated with MAMCs (E and F) stained for decorin (A, C and E) and versican (B, D and F). Bars, 100 μm.

**Figure 4:**
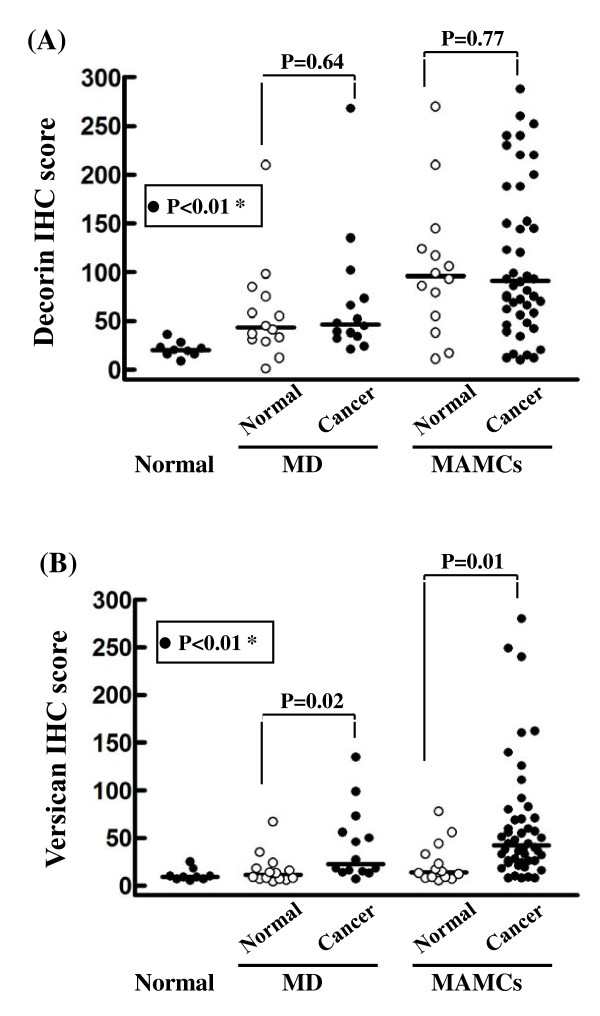
**Elevated expression of versican associates with MD and MAMCs in breast carcinomas**. Correlation of decorin (A) and versican (B) immunostaining in normal breast tissue, and normal and carcinoma tissues with mammographically detected high MD and MAMCs. Immunohistochemical (IHC) score was estimated as described in materials and methods. Bars represent the median value within each category. Differences between sub-groups (normal and cancer tissues with identical mammographic findings) were analyzed by Mann-Whitney *U*-test. (*) Statistically significant accumulation of decorin (A) and versican (B) in breast carcinomas associated with high MD and MAMCs [correlation of sub-groups: normal breast tissue, breast carcinoma with MD and breast carcinoma with MAMCs marked with black dots (•) using Kruskal-Wallis Anova].

In normal breast, staining for decorin was observed in the interstitial connective tissue surrounding the glands (Figure 2A), whereas negligible deposits of versican were also identified in stroma surrounding normal glands (Figure 2B). Interestingly, there was a marked stromal decorin and versican accumulation surrounding intraductal epithelial proliferations and *in situ *tumor components (Figure 2C and 2D). This was more diffusely distributed in the case of decorin, whereas versican staining was limited to the immediate proximity of the basement membrane. Prominent immunostaining for versican and decorin was observed in stroma associated with malignant areas of sectioned breast tissue. In the majority of patients with breast cancer, increased staining for versican and decorin was found, in the peritumoral and intratumoral stroma. In the central areas of malignant tumors, the staining was generally strong for both decorin and versican. The staining intensity for both proteoglycans was strong but varied considerably at the periphery of the same tumors. The expression of decorin and versican differed greatly in carcinomas with increased MD (Figure 3A and 3B) and carcinomas with MAMCs (Figure 3C and 3D). The expression of decorin was markedly elevated in breast carcinomas and normal tissues that are characterized by increased MD and MAMCs (Figure 3A, C, E and 4A). The overall expression of decorin was significantly higher in carcinomas and normal tissues with MAMCs (median ± SE: 91.5 ± 13.6 for carcinomas and 96.0 ± 18.9 for normal tissues) compared to tissues with increased MD (median ± SE: 46.5 ± 17.4 for carcinomas and 43.0 ± 13.7 for normal tissues) and normal tissue (median ± SE: 20.5 ± 2.5) (Figure 4A). No statistically significant differences were observed between sub-groups of carcinoma and normal tissues characterized by the presence of MD and MAMCs, indicating that decorin accumulation in the stroma may not correlate specifically with malignant transformation (Figure 4A). In contrast, the expression of versican was significantly higher only in carcinomas showing increased MD and MAMCs compared to normal tissues with identical mammographic findings (Figure 3B, D, F and 4B). The overall expression of versican was significantly higher in carcinomas with MAMCs (median ± SE: 42.0 ± 9.1 for carcinomas and 14.0 ± 5.8 for normal tissues) compared to tissues with increased MD (median ± SE: 22.5 ± 10.1 for carcinomas and 11.0 ± 4.4 for normal tissues) and normal tissue (median ± SE: 10.0 ± 2.0) (*P *< 0.01) (Figure 4B).

### Correlation between decorin and versican expression and tumor characteristics

**Figure 5:**
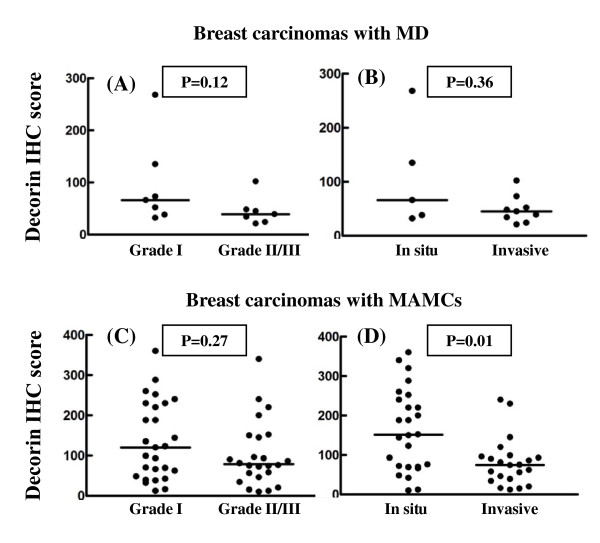
**Accumulation of decorin correlates with *in situ *breast carcinomas in patients with MAMCs**. Correlation between decorin immunostaining and tumor grade (A and C) and invasiveness (B and D) in breast carcinoma with high MD (A and B) and MAMCs (C and D). Differences between sub-groups were analyzed by Mann-Whitney *U*-test.

**Figure 6:**
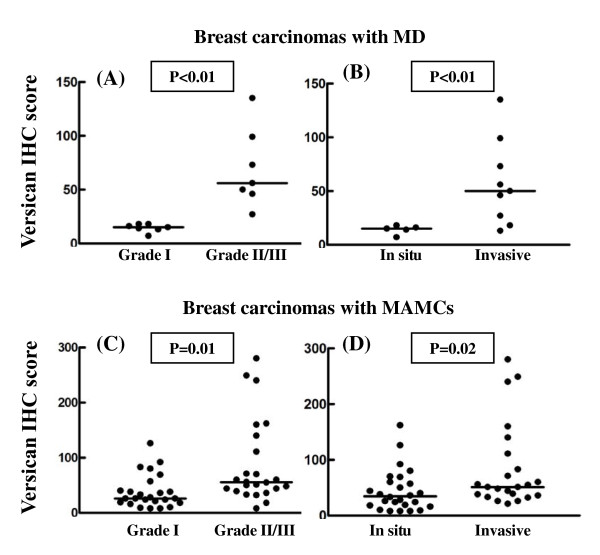
**Accumulation of versican correlates with higher tumor grade and invasive disease in breast carcinomas**. Correlation between versican immunostaining and tumor grade (A and C) and invasiveness (B and D) in breast carcinoma with high MD (A and B) and MAMCs (C and D). Differences between sub-groups were analyzed by Mann-Whitney *U*-test.

**Figure 7:**
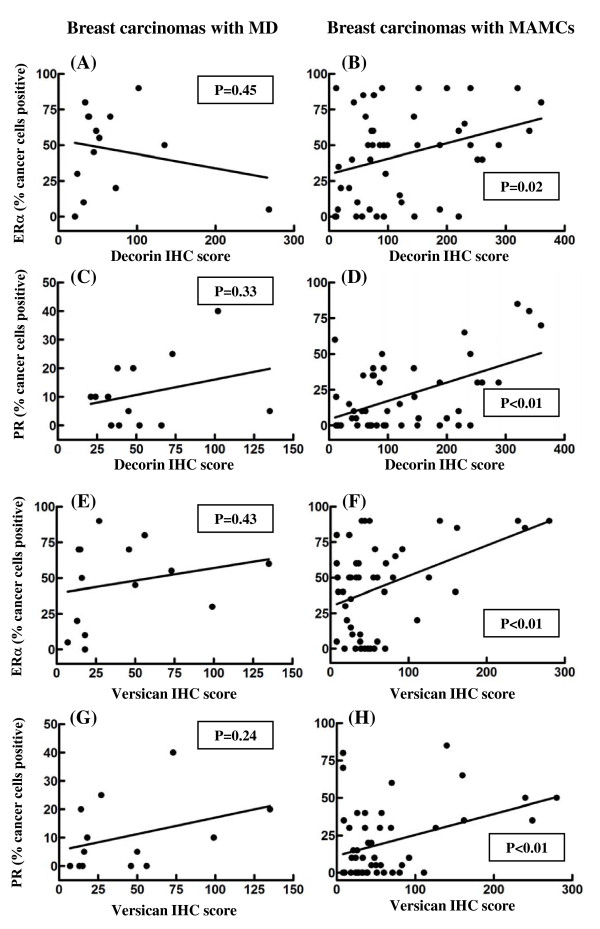
**Elevated expression of decorin and versican associates with higher expression of ERα and PR in breast carcinomas with MAMCs**. Correlation between decorin (A-D) and versican (E-H) immunohistochemical score (IHC) and percentage of tumor cells positive for ERα (A, B, E and F) and PR (C, D, G and H) in breast carcinoma with high MD (A, C, E and G) and MAMCs (B, D, F and H). Two-sided *P*-values were obtained by linear regression analysis (Pearson correlation).

Higher expression of decorin in the tumor stroma was found to associate with *in situ *breast cancer only in patients with MAMCs (*P *= 0.01) (Figure 5A-D). On the other hand the elevated expression of versican in the tumor stroma was strongly correlated with higher tumor grade and invasiveness in patients with MD and MAMCs (Figure 6A-D). The accumulation of decorin was significantly related to higher expression of ERα (*P *= 0.02) and PR (*P *< 0.01) in tumor cells only in patients with MAMCs (Figure 7A-D). Similarly, the expression of versican is significantly associated with higher expression of ERα (*P *< 0.01) and PR (*P *< 0.01) in tumor cells only in patients with MAMCs (Figure 7E-H). c-erbB2 expression was found in 1/14 carcinoma with MD and 13/48 of patients with MAMCs. The expression of both proteoglycans was not related to the presence of c-erbB2 in tumor cells in patients with MD and MAMCs (figure not shown).

## Discussion

An improved understanding of factors influencing changes in MD and the formation of MAMCs would improve their value and practical application in risk assessment in non-palpable breast cancer. Although both MD and microcalcifications have been shown to be influenced by various parameters [7,8,12,13,39,40], the biological basis underlying these tissue alterations is largely unknown [41]. Studies have shown that both stromal architecture and composition can exert an important influence on normal epithelial homeostasis [16,17,42], and somatic mutations can be identified in the stromal compartment of breast tumors independently of mutations in the neoplastic epithelium [43,44]. These observations are in accordance with the concept that stromal alterations might not always be 'reactive' but might sometimes play an initial 'landscaping' role in breast carcinogenesis, as has been proposed for the colon [45]. Several studies have shown the relationship between increased MD and specific epithelial lesions [7,8,39,46], whereas others have also noted a close association with stromal changes and suggest that MD correspond more directly to alterations in stromal composition including decorin [8,38,40]. In this study, we found a significant accumulation of decorin in normal breast tissues that are characterized by increased MD and MAMCs. A similar increase in decorin expression was noticed to the tumor stroma in non-palpable breast carcinomas with high MD and MAMCs. Decorin directly binds to collagen and affects fibrillogenesis and fibril spacing that are important aspects for stromal architecture and properties [32,47]. Taken into consideration our data and the previous observations that increased MD is associated with higher collagen density and extended fibrosis [38], it is likely that the deposition of decorin in MD and MAMCs might be associated with tissue fibrosis and matrix deposition. Furthermore, the observation that decorin levels are significantly higher in *in situ *compared to invasive breast carcinomas with MAMCs suggests that up-regulation of decorin prevents tumor spread. This is consistent with the observation that decorin inhibits various signaling cascades of the ErbB family of receptor tyrosine kinases [32,47] and low levels of decorin expression are associated with poor outcome in primary invasive tumors [34].

In contrast, the expression of versican is specifically up-regulated only in breast tissues with high MD and MAMCs, which are associated with malignant transformation. The deposition of versican in tumor stroma is significantly higher in MAMCs, a mammographic finding that is associated with higher risk for breast cancer than increased MD alone. The accumulation of versican in non-palpable carcinomas is related to higher tumor grade and the presence of invasive disease in lesions of both MD and MAMCs. This supports further the notion of a positive role for versican in tumor growth and progression in breast cancer. The increased accumulation of versican in tumor stroma by mammary fibroblasts is correlated with relapse in women with node-negative breast cancer [21]. Additional evidences in early-stage prostatic cancer [18] and ovarian cancer [25] supports the view that versican deposition is associated with progression of disease. Versican is thought to be an anti-adhesion molecule and this activity resides in the G1 domain of versican [30-32]. The interaction of versican with several binding molecules such as hyaluronan and CD44 promotes expansion of the pericellular matrix [48]. These complexes increase the viscoelastic nature of the pericellular matrix, creating a highly malleable extracellular environment which supports a cell shape change necessary for cancer cell proliferation and migration [30-32]. Furthermore, versican could influence cell proliferation by acting as a mitogen itself through the epidermal growth factor (EGF) sequences in the G3 domain [30,32], whereas versican G3 domain appears to be important in local and systemic tumor invasiveness of human breast cancer affecting both tumor cell survival and spread but also angiogenesis [49].

In postmenopausal women, MD is directly associated with circulating levels of estradiol [50] supporting a role of circulating hormones in breast cancer risk. Furthermore, aromatase the key enzyme converting testosterone to estradiol is increased in mammographically dense tissue in both the stroma and epithelium as demonstrated by immunohistochemistry [51]. We found that the elevated expression of decorin and versican by stromal fibroblasts in non-palpable breast carcinomas with MAMCs is positively correlated with the higher expression of ERα and PR by tumor cells. The expression of estrogen receptors in tumor cells most likely associate with increased levels of estrogens that regulate the growth of tumor cells in hormone-dependent breast cancer. Estradiol has found to play a key role in the expression of proteoglycans in breast cancer cells via its action to ERα [52]. Published data demonstrate that the biosynthesis of both proteoglycans is positively regulated by estrogens in osteoblastic cells [53] and normal endometrial stromal cells [54]. Possibly estrogens mainly produced locally by stromal fibroblasts in early stages of breast carcinogenesis drive through paracrine action tumor growth and through autocrine mechanisms the biosynthesis of versican and decorin by themselves. The expression of these proteoglycans is also regulated by various growth factors and cytokines present in increased amounts in the microenvironment of breast cancer lesions [30-32].

MAMCs are suggested to be a consequence of an active secretory process by the tumor cells [55]. Malignant mammary cells could express matrix molecules that would create an appropriate microenvironment to trigger calcifications by hydroxyapatite formation [55]. Benign or proliferative breast disease is associated with the deposition of oxalate calcifications, whereas hydroxyapatite crystals are related to invasive breast cancer [55]. Proteoglycans bind to hydroxyapatite and are involved in the calcification process acting either as promoters or inhibitors [56,57]. Decorin and biglycan are involved in the mineralization process and are required for normal bone development [58]. One hypothesis for the role of decorin in mineralization is that its modulation of collagen fibrillogenesis in the extracellular matrix indirectly affects hydroxyapatite crystal growth [59]. Decorin over-expression is also associated with intracellular calcification in epithelial cells [60]. The intracellular calcification may be a potential origin for pathologic calcification in breast cancer. Hydroxyapatite crystals stimulate mitogenesis and up-regulate the production of a variety of matrix metalloproteinases in malignant mammary cells and fibroblasts that mediate the degradation of the basement membrane and the surrounding matrix thus leading to the formation of distant metastases[61-63].

A possible scenario for the involvement of both proteoglycans in breast cancer progression may involve the accumulation of both versican and decorin by stromal fibroblasts in response to estrogens, growth factors and cytokines. The accumulated versican in the tumor stroma supports tumor growth and metastasis. Decorin is directly involved in the formation of a collagenous rich stroma associated with MD and may together with versican facilitate the formation of hydroxyapatite microcalcifications. In this process, stromal fibroblasts may be also involved. They over-express versican and decorin that may promote intracellular calcification. Hydroxyapatite crystals together with growth factors produced by cancer cells are capable to trigger cancer and stromal cell proliferation and up-regulation of synthesis of matrix degrading enzymes facilitating cancer cell spread.

## Conclusion

The results of this study suggest that the accumulation of versican in the tumor stroma is correlated with high MD and the formation of MAMCs associated with malignant transformation. The highest accumulation of versican was found in MAMCs, a mammographic finding of higher risk for malignancy in non-palpable breast lesions. The over-expression of versican was also significantly associated with the elevated expression of ERα and PR in tumor cells, higher tumor grade and invasiveness. These data suggest a key role for versican in disease progression and its possible use as molecular biomarker in non-palpable breast cancer.

## List of abbreviations used

EGF: epidermal growth factor; DCIS: ductal carcinoma *in situ*; MAMCs: malignant-appearing microcalcifications; MD: mammographic density; PBS: phosphate buffer saline.

## Competing interests

The authors declare that they have no competing interests.

## Authors' contributions

SSS carried out the immunoassays, performed the statistical analysis and helped to draft the manuscript, VTL carried out and evaluated the immunohistochemistry and helped to draft the manuscript, PR performed the histological and immunohistochemical evaluation and combined the figures, ELK performed and evaluated the mammographic screening, KD evaluated the immunostainings and combined the figures, HPK participated in the design of the study and helped to draft the manuscript, NKK participated in the design of the study and helped to draft the manuscript, ADT conceived of the study, participated in its design and coordination in the statistical analysis and to draft the manuscript. All authors read and approved the final manuscript.

## Pre-publication history

The pre-publication history for this paper can be accessed here:

http://www.biomedcentral.com/1471-2407/11/314/prepub
